# Production and persistence of specific antibodies in COVID-19 patients with hematologic malignancies: role of rituximab

**DOI:** 10.1038/s41408-021-00546-9

**Published:** 2021-09-14

**Authors:** C. Cattaneo, V. Cancelli, L. Imberti, K. Dobbs, A. Sottini, C. Pagani, A. Belotti, A. Re, A. Anastasia, V. Quaresima, A. Tucci, J. A. Chiorini, H. C. Su, J. I. Cohen, P. D. Burbelo, G. Rossi, L. D. Notarangelo

**Affiliations:** 1 Hematology, ASST Spedali Civili, Brescia, Italy; 2 CREA (AIL Center for Hemato-Oncologic Research), Diagnostic Department, ASST Spedali Civili di Brescia, Brescia, Italy; 3 Laboratory of Clinical Immunology and Microbiology, National Institute of Allergy and Infectious Diseases, National Institutes of Health, Bethesda, MD USA; 4 National Institute of Dental and Craniofacial Research, NIH, Bethesda, MD USA; 5 Laboratory of Infectious Diseases, NIAID, NIH, Bethesda, MD USA

**Keywords:** Antibodies, Haematological cancer

## Abstract

The ability of patients with hematologic malignancies (HM) to develop an effective humoral immune response after COVID-19 is unknown. A prospective study was performed to monitor the immune response to SARS-CoV-2 of patients with follicular lymphoma (FL), diffuse large B-cell lymphoma (DLBCL), chronic lymphoproliferative disorders (CLD), multiple myeloma (MM), or myelodysplastic/myeloproliferative syndromes (MDS/MPN). Antibody (Ab) levels to the SARS-CoV-2 nucleocapsid (N) and spike (S) protein were measured at +1, +3, +6 months after nasal swabs became PCR-negative. Forty-five patients (9 FL, 8 DLBCL, 8 CLD, 10 MM, 10 MDS/MPS) and 18 controls were studied. Mean anti-N and anti-S-Ab levels were similar between HM patients and controls, and shared the same behavior, with anti-N Ab levels declining at +6 months and anti-S-Ab remaining stable. Seroconversion rates were lower in HM patients than in controls. In lymphoma patients mean Ab levels and seroconversion rates were lower than in other HM patients, primarily because all nine patients who had received rituximab within 6 months before COVID-19 failed to produce anti-N and anti-S-Ab. Only one patient requiring hematological treatment after COVID-19 lost seropositivity after 6 months. No reinfections were observed. These results may inform vaccination policies and clinical management of HM patients.

## Introduction

COVID-19 carries an increased risk of death in patients with cancer, in particular for those with hematologic malignancies (HM), with mortality rates up to 40% among hospitalized patients [1–4].

In the absence of treatments of proven efficacy, vaccination could be a viable resource to prevent infection or reduce the risk of severe disease. However, patients with HM often manifest impaired innate and adaptive immunity, which may theoretically compromise the response to SARS-CoV-2 infection. Therefore, characterizing the quality, strength, and durability of adaptive immune responses to SARS-CoV-2 infection in patients with HM as compared with the general population would be of fundamental importance.

For most acute viral infections, neutralizing antibodies (Ab) rapidly rise after infection due to a burst of short-lived Ab-secreting cells, and then decline before reaching a stable plateau that can be maintained for years to decades by long-lived plasma and memory B cells [5]. Data on the dynamics of neutralizing Ab in the general population in the months following recovery from SARS-CoV-2 are limited; however, after an initial peak within 30 days, a progressive decline in the subsequent months has been observed, although neutralizing Ab is still detectable after 5 to 10 months [6–13]. Limited data are available regarding the immunological landscape of COVID-19 in HM patients. A recent study [14] showed significantly decreased percentages of classical monocytes, immunoregulatory NK cells, double-positive T cells, and B cells, when compared to COVID-19 patients without HM. Another study [15] reported a protective effect of high CD8 + cell counts on COVID-19 related mortality, irrespective of the concomitant presence of B-cell deficiency. On the other hand, hypogammaglobulinemia was negatively associated with the production of anti-SARS-CoV-2 IgG Ab in patients with chronic lymphocytic leukemia [16]. Furthermore, we previously reported that immunocompromised individuals have a delayed Ab response to the virus, compared with immunocompetent subjects [17]. At present, the long-term prognosis of HM patients surviving the acute phase of COVID-19 and their ability to develop and maintain robust specific Ab responses despite the need for immunosuppressive treatments are unknown. To elucidate this point, we performed a prospective longitudinal study of HM patients who had been hospitalized with COVID-19 and monitored their clinical outcome and immune response to SARS-CoV-2 in relation to both their underlying hematologic disease and the treatment received. Here we report data on the levels of anti-SARS-CoV-2 Ab during the first 6 months of convalescence.

## Subjects and methods

This was a prospective observational study, performed at the Hematology Department of ASST-Spedali Civili in Brescia, Italy, on patients with HM followed longitudinally after hospitalization during the acute phase of COVID-19. The study was conducted according to the principles of the Declaration of Helsinki, and was approved by the local Ethical Committee (protocol NP4156).

Patients affected by follicular lymphoma (FL), diffuse large B-cell lymphoma (DLBCL), chronic lymphoproliferative disorders (CLD), multiple myeloma (MM), myelodysplastic/myeloproliferative syndromes (MDS/MPN) who survived the acute phase of molecularly proven COVID-19 were eligible. Levels of specific Ab against the SARS-CoV-2 nucleocapsid (N) and spike (S) antigens were evaluated at 1 month (M1), 3 months (M3), and 6 months (M6) after documentation of a negative nasal swab for SARS-CoV-2 PCR. Nasal swabs were performed starting at least 2 weeks after the first documentation of a positive nasal swab for SARS-CoV-2 as determined by PCR; in case of persistence of positive nasal swab for SARS-CoV-2 PCR, it was repeated every week until PCR-negative.

After the acute phase of COVID-19, patients were managed at the Hematology Department as appropriate for their HM. Data about diagnosis, phase of hematologic disease, treatment received before and after COVID-19, time in which the swab became negative, and severity of SARS-Cov-2 infection were collected. The criteria and definitions for clinical classification of COVID-19 (mild, moderate, severe, and critically ill) were based on Guidance for Coronavirus Disease [18]. Specific Ab levels were measured at the same timepoints also in 18 individuals without hematologic disorders, selected among healthcare personnel from the same institution who had contracted COVID-19 during the same period of time, who served as controls.

### Analysis of SARS-CoV-2-specific Ab responses

Ab to the SARS-CoV-2 N and S proteins was measured using a fluid-phase luciferase-immunoprecipitation assay (LIPS), as previously described [17]. The LIPS assay has demonstrated high sensitivity and a wide dynamic range for Ab detection [17]. The cutoff for positivity of the tests were 125,000 light units (LU) for N Ab and 45,000 LU for S-Ab, respectively. All assays were performed at the NIH, Bethesda, USA by a scientist who was blinded to whether samples were from patients or controls.

### Statistical analysis

Patient’s characteristics were analyzed by standard descriptive statistics. The Student’s *t* test was used to compare continuous values; the Fisher’s exact test was used to compare differences in percentage. *P* values below 0.05 were considered statistically significant.

## Results

### Characteristics of patients

Forty-five patients with HM and SARS-CoV-2 infection were enrolled along with 18 patients with the infection without HM (controls). Median time from SARS-CoV-2 detection to SARS-CoV-2 PCR-negative nasal swabs in the patients was 30 days (range 8–81) and was not influenced by sex, age, hematologic diagnosis, disease status, the severity of SARS-CoV-2 infection, nor treatment received. All patients were tested for SARS-CoV-2 Ab at M1; 41 patients were tested at M3 (2 patients deceased, 1 refused, 1 was lost to follow-up) and 31 at M6 (5 refused and 5 were lost to follow-up). Hematological diagnoses included FL (9), DLBCL (8), CLD (8), MM (10), and MDS/MPS (10). Twenty-two patients (49%) were on active hematological treatment within 6 months before COVID-19 diagnosis. Fourteen patients had received immunochemotherapy prior to developing COVID-19; of these, 5 patients had received therapy >6 months, and 9 patients >6 months before SARS-COV-2 infection.

Eleven patients and 13 controls did not require any specific treatment for COVID-19; all the remaining patients and controls were treated with hydroxychloroquine/steroids/antivirals (lopinavir/ritonavir or darunavir/ritonavir). None of them received remdesivir, monoclonal antibodies, or convalescent plasma. Two patients required mechanical ventilation in ICU, while none of the controls were transferred in ICU.

Table 1 summarizes the clinical characteristics of patients and controls enrolled.

**Table 1:** Characteristics of patients and controls enrolled.

	Patients	Controls
M/F	26/19	7/11
Median age, y (range)	69 (35–85)	54 (33–66)
*Diagnosis*		*/*
FL	9	/
DLCL	8	/
CLD	8	/
MM	10	/
MDS/MPN	10	/
*On hematological treatment*^*a*^	*22*	*/*
On immunochemotherapy	9	
*Previous immunochemotherapy*^*b*^	*5*	*/*
Hypogammaglobulinemia (IgG < 400 mg/dl)	7	/
*Severity of COVID disease*		
Mild/moderate	18	14
Severe	25	4
Critical	2	/
*Admission at hospital*	42	4
*Treatment of COVID-19 received*		
None	11	13
Steroids	31	5
Hydroxychloroquine	34	4
Antivirals^c^	20	/
ICU admission	2	/
SARS-CoV-2→swab negativity, median days (range)	30 (8–81)	19 (9–31)

^a^Within 6 months before COVID diagnosis.

^b^More than 6 months before COVID diagnosis.

^c^Lopinavir/ritonavir or darunavir/ritonavir.

*FL* follicular lymphoma, *DLCL* diffuse large cell lymphoma, *CLD* chronic lymphoproliferative disorders, *MM* multiple myeloma, *MDS/MPS* myelodysplastic/myeloproliferative syndromes, *ICU* intensive care unit.

### Humoral response in hematologic patients

Mean levels of anti-N and anti-S-Ab levels after 1, 3, and 6 months from documented SARS-CoV-2 PCR-negative nasal swabs were measured in patients with HM and controls (Table 2). Anti-N-Ab levels did not differ significantly in patients and in controls except at M1, when they were higher in HM patients (1832891 LU vs 826820 LU, *p* = 0.016).

**Table 2:** Mean levels (LU) of anti-N and anti-S-Ab in patients and controls (all samples).

	M1, mean levels (CI)	M3, mean levels (CI)	M6, mean levels (CI)
Anti-N-Ab (patients)	1,832,891 (1,371,900–2,293,900)	1,609,252 (112,700–2,005,800)	500,246 (273,325–727,167)
Anti-N-Ab (controls)	826,820 (550,564–1,103,100)	922,600 (550,275–1,294,900)	558,221 (−982–1,174,000)
Anti-S-Ab (patients)	956,272 (654,366–1,258,200)	986,785 (640,477–1,333,100)	790,246 (351,550–1,229,700)
Anti-S-Ab (controls)	677,174 (200,799–1,153,600)	859,891 (527,787–1,192,000)	693,385 (206,899–1,179,000)

*Anti-N-Ab* anti-nucleocapsid antibodies, *Anti-S-Ab* anti-spike antibodies, *M1* +1 month timepoint, *M3* +3 month timepoint, *M6* +6 month timepoint, *CI* confidence interval.

**Fig. 1:**
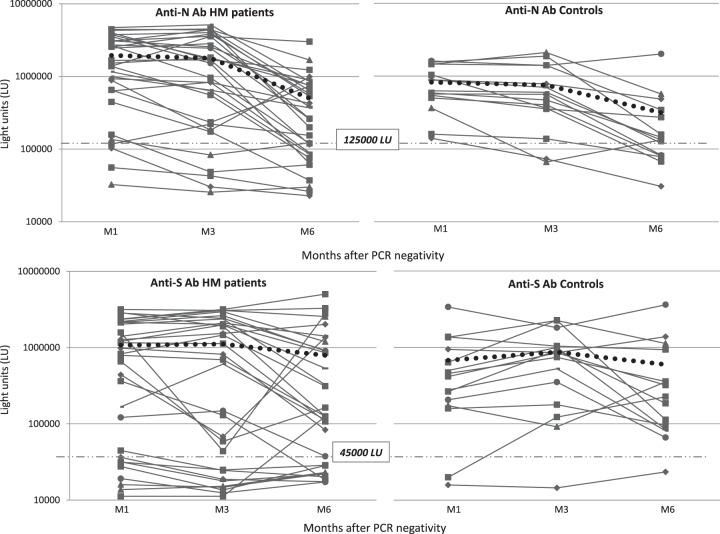
Longitudinal profile of antibodies against nucleocapsid and spike protein in patients with and without hematological malignancy. Nucleocapsid antibody (anti-N-Ab) levels and spike antibody (anti-S-Ab) levels were determined at 1 (M1), 3 (M3), and 6 (M6) months after nasal swabs became PCR-negative. The levels of Ab in light units against the nucleocapsid (top two panels) and spike protein (bottom two panels) over time were plotted on the *y* axis. The mean values of Ab levels are represented by the thick dotted lines. The cutoff values for determining seropositivity for anti-N-Ab and anti-S-Ab are shown by the thin dashed lines.

Considering the 31 patients for whom specific Ab levels were available at all timepoints, anti-N-Ab levels remained relatively stable at M3 and declined at M6 both in patients and in controls (controls: M1 vs M3 826820 vs 754197 LU, *p* = 0.35, M3 vs M6 754197 vs 313989 LU, *p* = 0.014; patients: M1 vs M3 1946181 vs 1779786 LU, *p* = 0.33, M3 vs M6 1779786 vs 500246 LU, *p* = 0.000037). Mean anti-S-Ab levels did not differ between patients and controls at M1 and remained stable over time both at M3 and M6 (controls: M1 vs M3 677174 vs 866734 LU, *p* = 0.3, M3 vs M6 866734 vs 596646 LU, *p* = 0.25; patients: M1 vs M3 1073640 vs 1112817 LU, *p* = 0.3, M3 vs M6 1112817 vs 790613 LU, *p* = 0.1) (Fig. 1).

A wide range of Ab levels was detected in patients compared with controls. In particular, the percentage of subjects with detectable Ab levels was lower in HM patients both for anti-N and anti-S-Ab and at all time points. Among control subjects, 100% had developed anti-N-Ab at M1, which were still detectable in 89% of subjects at M3 and 65% at M6. Anti-S-Ab were detectable in all but two controls at M1 and in all but one at M3 and M6. By contrast, among HM patients the percentage of seropositive subjects for anti-N-Ab varied from 80% at M1 to 78% at M3 and 61% at M6, and the proportion of anti-S seropositive patients was 71% at M1, 66% at M3, and 68% at M6. These differences in anti-S-Ab responses between HM patients and controls were significant at M3, when 27/41 patients (66%) compared with 17/18 controls (94%) (*p* = 0.02) had detectable anti-S-Ab, and were borderline at M6, when the persistence of anti-S-Ab was detected in 21/31 patients (68%) compared with 16/17 controls (94%) (*p* = 0.07).

### Humoral response according to hematologic diagnosis and treatment

Patients with lymphoma showed a lower Ab response compared with patients with other HM at any time point. In particular, anti-N-Ab were detected at lower frequency in patients with FL and DLBCL than in those with MM, CLD and MDS/MPN [M1: 10/17 (59%) vs 26/28 (93%), *p* = 0.017; M3: 10/16 (63%) vs 22/25 (88%), *p* = 0.12; M6: 5/12 (42%) vs 14/20 (70%), *p* = 0.15]. Similar results were obtained for anti-S-Ab [M1: 8/17 (47%) vs 24/28 (86%), *p* = 0.008; M3: 7/16 (44%) vs 20/25 (80%), *p* = 0.023; M6: 6/12 (50%) vs 15/20 (75%), *p* = 0.25].

**Fig. 2:**
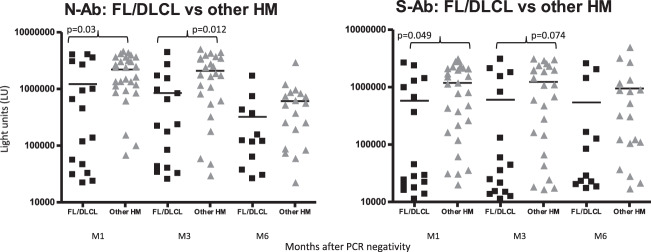
Antibody levels against SARS-CoV-2 nucleocapsid and spike protein in hematologic patients with COVID-19. Antibody levels against SARS-CoV-2 nucleocapsid (NAb) and spike protein (S-Ab) were determined among the follicular lymphoma (FL) and diffuse large B-cell lymphoma (DLCL) compared with patients with other hematological malignancies. Each symbol represents an individual patient sample from 1 (M1), 3 (M3), and 6 (M6) months after nasal swabs became PCR-negative. Antibody levels are plotted in light units (LU) on the *y* axis and the solid horizontal line represents the mean level for each group. Statistically significant differences in antibody levels among the sample group were determined by a Student’s *t* test.

Mean anti-N and anti-S-Ab levels in patients with FL and DLBCL were also lower than those observed in patients with other HM at M1 (anti-N: 1217517 vs 2205610 LU, *p* = 0.03; anti-S: 580444 vs 1184453 LU, *p* = 0.049), M3 (anti-N: 850510 vs 2094487 LU, *p* = 0.012; anti-S: 605284 vs 1230946 LU, *p* = 0.074) and M6 (anti-N: 323314 vs 612456 LU, *p* = 0.21; anti-S: 540220 vs 722794 LU, *p* = 0.36) (Fig. 2).

Only 1 of 10 patients affected by MDS/MPS did not show seroconversion in N and S antigens at any time point, and none of MM patients showed Ab levels below the positivity cutoff at any time point, except for one patient, who became negative at M6.

**Fig. 3:**
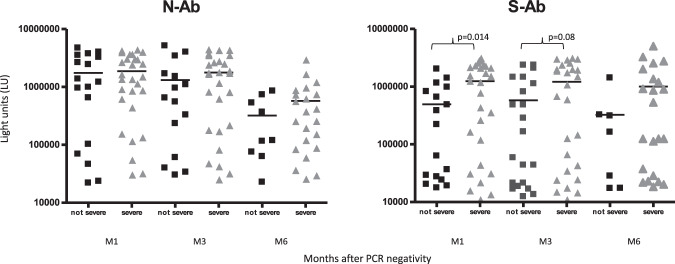
Correlation of antibody levels against SARS-CoV-2 nucleocapsid and spike protein with patient severity. Antibody levels against SARS-CoV-2 nucleocapsid (N-Ab) and spike protein (S-Ab) were determined in the hematological cohort based on the clinical severity scale, severe vs non-severe, based on the criteria described in the material and methods. Each symbol represents an individual patient sample from a given timepoint. Antibody levels are plotted in light units on the *y* axis and the solid horizontal line represents the mean level for each group. Statistically significant differences in antibody levels among the sample group were determined by a Student’s *t* test.

Neither the time to have a negative swab, nor hypogammaglobulinemia (IgG levels <400 mg/dl) which were present in 7/45 patients, had an impact on levels of anti-N and anti-S-Ab (Fig. S1) nor on seroconversion rates (Table S1). The severity of SARS-CoV-2 infection did not impact on anti-N and anti-S-Ab positivity rates nor on mean levels of anti-N-Ab; however, patients with severe or critical COVID-19 showed higher levels of anti-S-Ab than those with mild/moderate disease, particularly at M1 (*p* = 0.014) (Fig. 3).

### Impact of rituximab on humoral response

**Fig. 4:**
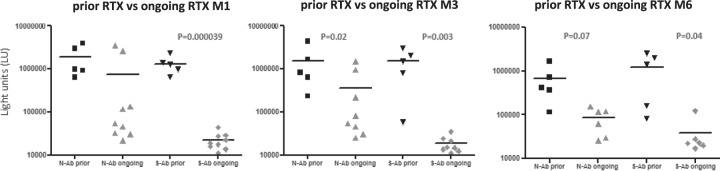
Antibody levels against SARS-CoV-2 nucleocapsid and spike proteins in patients receiving prior or ongoing Rituximab treatment. Antibody levels against SARS-CoV-2 nucleocapsid (N-Ab) and spike protein (S-Ab) were determined in hematological patients receiving prior or ongoing Rituximab treatment. Comparison of antibody levels in these two groups was determined at 1 (M1), 3 (M3), and 6 (M6) months after nasal swabs became PCR-negative. Statistically significant differences in antibody levels among the sample group were determined by a Student’s *t* test.

**Fig. 5:**
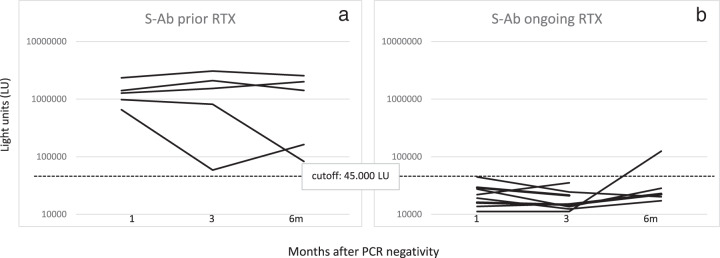
Longitudinal profile of antibodies against the spike protein in hematological patients receiving prior or ongoing rituximab treatment. Spike antibody (S-Ab) levels were determined at 1, 3, and 6 months after nasal swabs became PCR-negative in hematological patients receiving prior (**a**) or ongoing rituximab treatment (**b**). The cutoff value for determining seropositivity for S-Ab is shown by the dotted lines.

Rituximab (RTX) had been administered to 14/17 NHL patients before they developed COVID-19. In particular, five of these patients had received RTX ≥ 6 months (prior RTX group), and nine patients <6 months (ongoing RTX group) before COVID-19 diagnosis. Levels of anti-N and anti-S-Ab were lower in the “ongoing RTX” than in “prior RTX” group. This difference was more pronounced for anti-S-Ab, being statistically significant at all timepoints (M1: 22317 vs 1332020 LU, *p* = 0.000039; M3: 18500 vs 1515058 LU, *p* = 0.003; M6: 43178 vs 1245654 LU, *p* = 0.04) (Fig. 4). None of the nine patients in the “ongoing RTX” group had seroconverted at M1 compared with 5/5 in the “prior RTX” group (*p* = 0.0005). No changes occurred in the rate of seropositive patients at M3 and M6 except for one patient in the “ongoing RTX” group who seroconverted at M6 (Fig. 5).

Among eight patients with CLD, only one was receiving RTX monotherapy for concomitant hemolytic anemia at the time of COVID-19 diagnosis; this patient failed to mount anti-S-Ab at the time points (M1 and M3) serological studies were performed.

### Clinical follow-up

Sixteen patients required therapy for their HM during follow-up; they are described in detail in Table S2. Fourteen of them were evaluable at all timepoints; only one lost seropositivity at M6 after completing 6 courses of R-CHOP. Seropositivity was maintained over time in all the Controls. Twenty-eight patients did not require therapy during follow-up; among these, only one, affected by MM, lost seropositivity at M6. No cases of SARS-CoV-2 reinfection were observed.

Nine patients showed progression or relapse of their hematologic disease and two of them (one MDS/MPN, one MM) rapidly died after M1. Ab titers persisted in six of the other seven patients during the entire follow-up period.

## Discussion

The persistence over time of humoral immune responses to SARS-Cov-2 after COVID-19 is a topic of major clinical and epidemiologic relevance. In non-immunocompromised individuals, Yamayoshi et al. [19] documented kinetics of Ab production typical of other viral infections and Ab persistence for at least 90 days in a small cohort of patients, whereas in a similar cohort Crawford et al. [20] confirmed kinetics of Ab titers consistent with the expected early immune response to viral infection, but a progressive decline between 30 and 152 days after symptoms onset. Similar results concerning the persistence of Ab detection have also been reported by Seow et al. [6]. Several studies have documented that seroconversion occurs after 11–20 days in immunocompetent individuals [21–23]. Moreover, higher levels of anti-S-Ab have been reported in patients with severe or critical disease than in those with mild or moderate symptoms [9, 13, 22, 24]. In two large population studies in Iceland and in New York no reduction in neutralizing Ab was noted over a follow-up of 4 and 5 months respectively [8, 25], and more recent studies have shown neutralizing Ab still detectable up to 10 months [11–13].

Humoral immune responses have not been studied extensively in COVID-19 patients with comorbidities, and with HM in particular. O’Nions et al. [26] reported nine patients with different types of acute leukemia whose Ab response to SARS-CoV-2 was somewhat delayed but otherwise similar to non-immunocompromised subjects. On the other hand, in a series of 21 CLL patients, a seroconversion rate of 67% and a delay in the time of Ab responses were reported [16]. In another study, among 12 patients with HM whose Ab responses to SARS-CoV-2 were measured on average 13 days after symptom onset, only two showed evidence of seroconversion [27]. We have previously reported blunted SARS-CoV-2-specific Ab responses in three immunocompromised individuals, including one with CLL [17]. A marked defect of the humoral immune response in hematologic patients compared with solid cancer patients and normal individuals were reported by Huang et al. [15], who underscored the alternative protective effect of an adequate cellular response by CD8+ lymphocytes. However, the kinetics of the immune response in HM over time has not been reported in the above studies. Furthermore, very little is known on the impact of active chemotherapy on immunological memory to SARS-CoV-2 infection. In this regard, it is interesting that Liu et al. [28] have documented reversal of class-switched responses (from IgG to IgM) in one patient with acute leukemia.

In our prospective longitudinal study, we analyzed the capacity of HM patients to produce adequate levels of anti-S and anti-N-Ab after SARS-CoV-2 infection, and to maintain them over time, up to 6 months after nasal swabs for SARS-CoV-2 PCR swab became negative, i.e., about 7 months after infection. In fact, in our series, the median time from diagnosis to PCR negativity was 30 days, which was higher than 19 days in controls, an interval consistent with what was previously reported in immunocompetent individuals [29, 30], indirectly confirming that HM patients have a blunted acute immune response to SARS-CoV-2 infection.

At one month after COVID-19 PCR-negative nasal swabs, serological responses to SARS-CoV-2 were comparable in HM patients and in normal controls, and titers of anti-N-Ab were even higher in HM patients. The greater severity of clinical manifestations in patients than in controls may be responsible for the higher N-Ab levels in patients, as it is known that the levels of Ab are higher in patients with a more severe form of the disease [13]. Furthermore, no differences in mean Ab levels between HM patients and controls were observed both for anti-N and for anti-S-Ab at 3 and 6 months, indicating that in general patients with HM are capable of mounting a robust humoral immune response lasting at least several months. A similar decline in anti-N-Ab levels was observed both in HM patients and in controls, after 6 months, consistent with previous observations in immunocompetent individuals [6]. However, patients with HM showed a wide range of Ab levels, and ~one-third of these patients failed to seroconvert at the one-month time point. This finding is entirely owing to the different behavior of patients with FL and DLBCL, who showed the lowest values of mean anti-S-Ab levels and a seroconversion rate at M1 lower than 50%, whereas patients with MDS/MPN, MM, and CLD had seroconversion rates of at least 85%, similar to normal controls. In both groups, no significant changes in the level of anti-S-Ab and in the proportion of seropositive individuals were observed after 3 and after 6 months.

A more careful analysis of the subgroup of lymphoma patients revealed that the main factor accounting for the lack of serological response was treatment with RTX. In fact, patients receiving RTX within 6 months from the diagnosis of COVID-19 showed an impaired humoral response, in particular for anti-S-Ab. RTX treatment has been previously associated with defective humoral responses in HM patients [31]; in our study, we demonstrated that recent use of RTX abolished anti-SARS-CoV-2 humoral responses for at least 6 months after recovery from infection, in the vast majority of patients treated. The causal role of treatment rather than of the hematological diagnosis is reinforced by the appropriate humoral response observed in patients with MM or CLD, two conditions where a perturbation of the humoral immune response should also be expected. Moreover, the single patient with CLD who had received RTX, also failed to develop anti-SARS-Cov-2 antibodies.

Despite their severely impaired humoral response, RTX-treated NHL patients surviving COVID-19 did not experience a higher rate of infections nor of other complications during the follow-up period. Indeed, B-cell depletion and a defective humoral immune response may have a limited impact on clinical outcome, as suggested by a large epidemiological Italian study where the fatality rate of acute COVID-19 was similar among patients with myeloid neoplasms, plasma cell neoplasm, or lymphoma [1]. Furthermore, good outcomes after SARS-CoV-2 infection have been reported in patients with X-linked agammaglobulinemia (XLA), who lack B cells and therefore are unable to mount specific ab responses [32, 33]. However, whether these patients can mount memory T-cell responses that are sufficient to confer long-term protection, remains to be seen. A possible case of SARS-CoV-2 reinfection in a patient with XLA has been recently reported [34], raising concerns on the role of T-cell responses in patients with defective Ab production. These considerations have an impact on the implementation of immunization strategies for patients with HM who remain severely B-cell lymphopenic after treatment with RTX. In our study, the single patient treated with a Bruton’s tyrosine kinase inhibitor showed only anti-N-Ab positivity for 3 months.

Our study has several limitations. The number of patients included was relatively small. Therefore, our findings need to be interpreted with caution and must be confirmed in larger cohorts. Moreover, the patients included in the study had been all hospitalized for COVID-19; therefore, no conclusions can be drawn for HM patients experiencing asymptomatic infection or mild COVID-19.

Nevertheless, our study provides reassuring data about the ability of HM patients to develop a persistent ab response to the S protein, with the exception of RTX-treated subjects, whose ability to generate specific Ab seems almost completely abolished for up to 6 months when this treatment is given within 6 months before infection, whereas patients who had received RTX more remotely apparently retain the ability to mount a humoral response. Bird et al. [35] reported only 56% of MM IgG Ab positive after SARS-CoV-2 vaccination; the percentage was even lower in CLL patients (39.5%) [36]. Although larger epidemiological studies are warranted to explore the efficacy of vaccination in HM patients, we believe that our study provides useful information that may inform vaccination policies to be adopted in these patients.

## Supplementary information

Supplemental material

aj-checklist
