# Supplementary material for: Production and persistence of specific antibodies in COVID-19 patients with hematologic malignancies: role of rituximab
Source: Blood Cancer J. 2021 Sep 14;11(9):151. doi: 10.1038/s41408-021-00546-9 (PMC8438656; doi:10.1038/s41408-021-00546-9)
Supplement: Supplementary file 1 — Supplemental material [file 41408_2021_546_MOESM1_ESM.docx]

**Table S1: Seroconversion rates according to serum Ig levels**

|  | M1 | | | M3 | | | M6 | | |
| --- | --- | --- | --- | --- | --- | --- | --- | --- | --- |
|  | **Ig≥400 mg/dl** | **Ig<400 mg/dl** | **P value** | **Ig≥400 mg/dl** | **Ig<400 mg/dl** | **P value** | **Ig≥400 mg/dl** | **Ig<400 mg/dl** | **P value** |
| Anti-N Ab positivity | 32/38 (84%) | 4/7 (57%) | 0.13 | 29/35 (83%) | 3/6 (50%) | 0.1 | 7/27 (63%) | 2/4 (50%) | 0.63 |
| Anti-S Ab positivity | 29/38 (76%) | 3/7 (43%) | 0.16 | 25/35 (71%) | 2/6 (66%) | 0.16 | 19/28 (70%) | 2/4 (50%) | 0.53 |

Anti-N Ab: anti-nucleocapsid antibodies; Anti-S Ab: anti-spike antibodies; M1: +1month timepoint; M3: +3month timepoint; M6: +6month timepoint

**Table S2: Anti-N and anti-S Ab positivity in patients receiving treatment for hematologic disease after Covid-19**

| Patient ID | Diagnosis | Treatment received after Covid-19 | Months after swab negativity | N-Ab positivity (M1-M3-M6) | S-Ab positivity (M1-M3-M6) |
| --- | --- | --- | --- | --- | --- |
| 1 | CLD | Acalabrutinib | 1 | +/+/- | -/-/- |
| 2 | DLCL | R-CHOP | 0 | +/+/- | +/+/- |
| 3 | DLCL | ASCT | 1 | -/-/- | -/-/- |
| 4 | FL | ASCT | 2 | +/+/- | -/-/- |
| 5 | FL | R-bendamustine | 3 | +/+/- | +/+/+ |
| 6 | MDS/MPS | Decitabine+venetoclax | 2 | -/-/- | -/-/- |
| 7 | MDS/MPS | Hydroxyurea | 0 | +/+/+ | +/+/+ |
| 8 | MDS/MPS | Hydroxyurea | 0 | +/NA/NA | +/NA/NA |
| 9 | MM | Bortezomib-DMZ | 4 | +/+/- | +/+/+ |
| 10 | MM | Bortezomib, Melphalan, PDN | 2 | +/+/- | +/+/+ |
| 11 | MM | Melphalan, prednisone | 3 | +/+/+ | +/+/+ |
| 12 | MM | Bortezomib, melphalan, DMZ | 1 | +/+/+ | +/+/+ |
| 13 | MM | Daratumomab, lenalidomide, DMZ | 1 | +/+/+ | +/+/+ |
| 14 | MM | Daratumomab, lenalidomide, DMZ | 1 | +/+/+ | +/+/+ |
| 15 | MDS/MPS | Hydroxyurea | 0 | +/+/+ | +/-/+ |
| 16 | MDS/MPS | Hydroxyurea | 0 | +/+/NA | +/+/NA |

N-Ab: anti-nucleocapsid antibodies; S-Ab: anti-spike antibodies; CLD: chronic lymphoproliferative disorders; FL: follicular lymphoma; DLCL: diffuse large cell lymphoma; MDS/MPS: myelodysplastic/myeloproliferative syndromes; MM: multiple myeloma; R: Rituximab; ASCT: autologous stem cell transplant; DMZ: dexamethasone; PDN: prednisone; NA: not available; M1: +1month timepoint; M3: +3month timepoint; M6: +6month timepoint

**Fig. S1: Mean anti-N and anti-S Ab levels according to the presence of hypogammaglobulinemia**

**
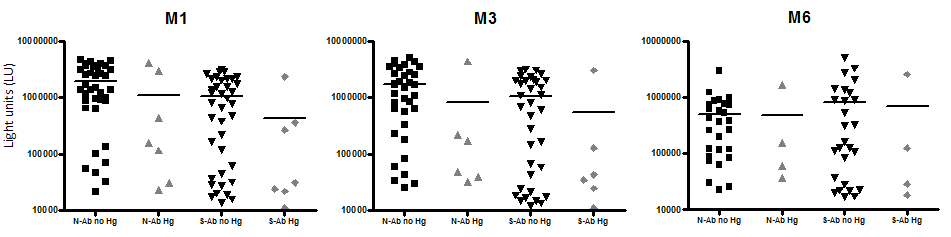
**

N-Ab: anti-nucleocapsid antibodies; S-Ab: anti-spike antibodies; Hg: hypogammaglobulinemia (i.e. Ig<400 mg/dl); M1: +1month timepoint; M3: +3month timepoint; M6: +6month timepoint
